# Supplementary material for: Compounds from plantar foot sweat, nesting material, and urine show strain patterns associated with agonistic and affiliative behaviors in group housed male mice, Mus musculus
Source: PLoS One. 2021 May 14;16(5):e0251416. doi: 10.1371/journal.pone.0251416 (PMC8121354; doi:10.1371/journal.pone.0251416)
Supplement: S1 File — (DOCX) [file pone.0251416.s001.docx]

# S1 File. Preparation of Ketone (4)

**General Procedure**

All reactions were performed in flame or oven-dried glassware under argon atmosphere unless otherwise noted. All commercial reagents were used as received unless otherwise noted. All materials were vacuum dried (1-5mmHg) to remove trace elements of solvent. “*in vacuo*” refers to bulk solvent removal which was performed by Buchi rotary evaporator linked to a water aspirator. Bulk solvent removal of solvents with boiling points above 80°C was performed on a Buchi rotary evaporator which was connected to Precision Scientific vacuum which allowed for pressures of 1 mmHg. Bulk grade solvents hexanes and ethyl acetate were distilled before use for chromatography. Diethyl ether (Et_2_O), tetrahydrofuran (THF), methylene chloride (CH_2_Cl_2_), dimethylfomamide (DMF), and toluene (tol) were dried on a commercial solvent system before use in reactions. Hexamethylphosphoramide (HMPA) and N, N’-dimethylpropyleneurea (DMPU) were both distilled from CaH_2_ and stored over 3 Å molecular sieves. Triethylamine (Et_3_N), pyridine (pyr) and diisopropylethylamine (DIPEA) were distilled from CaH_2_ under dry argon immediately before use.

Proton nuclear magnetic resonance (^1^H NMR) spectra and carbon nuclear magnetic resonance (^13^C NMR) spectra were measured on a Varian VXR (400MHz), Varian INOVA-400 (400MHz), Varian INOVA 500 (500MHz) insturments. H^1^ NMR and ^13^C NMR are reported in parts per million (ppm) downfield from tetramethylsilane and calibrated using residual undeuterated chloroform as an internal standard which is set to δ 7.26. ^1^H NMR spectra data were reported in the form δ (multiplicity, coupling constants (Hz), integration). Multiplicities are reported as follows: s = singlet, d = doublet, t = triplet, q = quartet, m = multiplet, br = broad, dd = doublet of doublet, dt = doublet of triplet, ABq = AB quartet. Mass spectra data (GCMS, LCMS, HRMS) were recorded on an Agilent technologies 6890N 15973 (EI), Agilent Technologies 1200 series/6130(ESI), and Waters/Synapt Horns mass spectrometers using chemical ionization (CI) with methane and / or electrospray ionization (ESI).

Analytical thin-layer chromatography (TLC) was performed using glass backed 0.25 mm thickness silica gel 60 (F_254_) plates which were visualized under UV light and/or by staining with ethanolic p-anisaldehyde, potassium permanganate, vanillin, dintrophenylhydrazine, and bromocresol green followed by heating on a hot plate. Iodine crystals were used to develop TLC plates in a glass chamber. Flash chromatography was performed using Merck silica gel 60 (Kiesegel 60) from Whatman Scientific or Sorbent Technologies and pressure was obtained using an in-house airline.

**Synthesis of 3,5-diethyl-2-hydroxycyclopent-2-en-1-one (4)**

**3-ethyl-2-methoxycyclopent-2-en-1-one (2)**

Acetone (20mL) from a freshly opened bottle was added into a 50 mL round bottom flask charged with commercially available ketone **(1)** (500mg, 4 mmol, 1 equiv.). Following solvation, K_2_CO_3_ (1.05g, 7.9 mmol, 2 equiv) was added and followed by dropwise addition of methyl iodide (0.5 mL, 7.9 mmol, 2equiv). The mixture was allowed to stir for 48 hours until all starting material was consumed. The reaction mixture was then concentrated *in vacuo* and diluted with diethyl ether and water (1:1). The aqueous phase was separated and extracted with diethyl ether (2x 20 mL). The organic phases were combined and then washed with brine distilled water followed by drying over anhydrous MgSO_4_, filtered and concentrated *in vacuo*. The remaining crude oil was purified by column chromatography (10% EtOAc: 90%Hex) to yield 515mg (92%) of Ketone **(2)** as a colorless oil; characterized by R_f_ 0.31 (20%EtOAc:80%Hex); ^1^H NMR (400MHz, CDCl_3_) δ 3.86 (s, 3H), 2.38 (m,6H), 1.12 (t,3H) ^13^C NMR (500MHz, CDCl_3_) δ203.77, 159.88, 152.16, 58.66, 33.12, 24.62, 22.14, 11.74. IR (thin film): 2928, 2868, 1703 cm^-1^ HRMS m/z [M]^+^ calcd for C_8_H_12_O_2_ 140.08, found 140.0828. Derived from J.Org.Chem 2019,84,7166-7174

**3,5-diethyl-2-methoxycyclopent-2-en-1-one (3)**

Ketone **(2)** (50mg, 0.36mmol) was added to 1.2 mL of dry THF and stirred at -78°C. A [0.5] molar solution of LDA (0.4mmol, 1 equiv) dissolved in THF was added dropwise followed by addition of 0.6 mL of DMPU. This solution was stirred at -78°C for 3 hours then ethyl iodide (37.8 µL, 0.47 mmol, 1.3 equiv) was added and the reaction was warmed to- 40°C with an acetonitrile/dry ice bath. After stirring for 6 hours at -40°C the reaction was slowly quenched at -40°C by dropwise addition of distilled water. The mixture was extracted with diethyl ether (2x10mL). The organic layers were combined and washed with brine and distilled water followed by drying over MgSO_4_, filtered, and concentrated *in vacuo*. The crude oil was purified by column chromatography (7% EtOAc: 93%Hex) to yield 45mg (77%, 94% brsm) of the diethyl ketone **(3)** as a translucent yellow oil and 10 mg of starting material **(2)**; characterized by R_f_ 0.47 (20%EtOAc:80%Hex); ^1^H NMR (400MHz, CDCl_3_) δ 3.86 (s, 3H), 2.38 (m,6H), 1.12 (t,3H); ^13^C NMR (500MHz, CDCl_3_) δ 205.95, 158.55, 151.59, 58.66, 44.93, 31.24, 24.62, 22.03, 11.81, 11.15; IR (thin film) 2926, 2875, 1703 cm^-1^; HRMS m/z [M]^+^ calcd for C_10_H_16_O_2_ 168.1145, found 168.1146.

**3,5-diethyl-2-hydroxycyclopent-2-en-1-one (4)**

A 25 mL double necked round bottom flask was fitted with a condenser and charged with the diethyl ketone **(3)** (50mg, 0.29 mmol, 1equiv) and 5mL of 4M aqueous HCl was added while stirring. The solution was heated to reflux for 90 minutes until all starting material was consumed. The mixture was diluted with 5 mL of distilled water and then extracted with diethyl ether. The organic layer was washed with brine and then distilled water. The organic phase was then separated and then dried over anhydrous MgSO_4_, filtered, and concentrated *in vacuo*. The crude material was purified by column chromatography (10% EtOAc: 90%Hex) to yield 40mg of ketone **(4)** (89%) as a colorless oil which later crystallized when stored at -20°C; characterized by R_f_ 0.36 (20%EtOAc:80%Hex); ^1^H NMR (400MHz, CDCl_3_) δ 3.86 (s, 3H), 2.38 (m,6H), 1.12 (t,3H); ^13^C NMR (500MHz, CDCl_3_) δ 205.71, 148.42, 147.68, 44.21, 31.57, 24.56,21.79, 11.41; IR(thin film): 3442, 2967, 1690 cm^-1^; HRMS m/z [M]^+^ calcd for C_9_H_14_O_21_ 154.09883, found 154.09882.
