# Supplementary figures and images for: Compounds from plantar foot sweat, nesting material, and urine show strain patterns associated with agonistic and affiliative behaviors in group housed male mice, Mus musculus
Source: PLoS One. 2021 May 14;16(5):e0251416. doi: 10.1371/journal.pone.0251416 (PMC8121354; doi:10.1371/journal.pone.0251416)

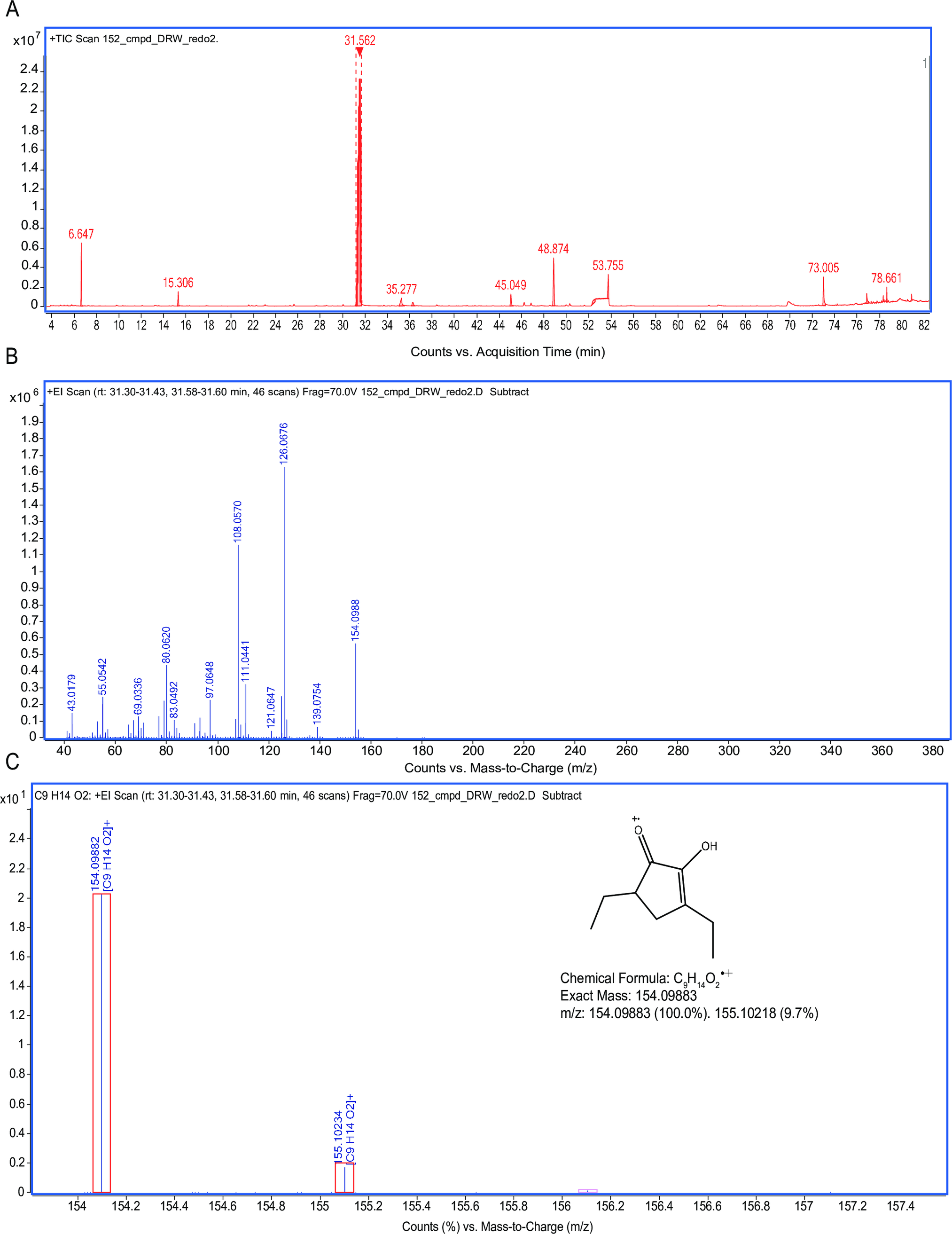

Supplement: S1 Fig — (A) Total ion chromatogram (TIC); peak elutes at 31.562 minutes. (B) Full mass spectrum of 31.562 minute peak. (C) Molecular ion region. The boxes represent the theoretical distribution. (TIF) [file pone.0251416.s002.tif]
