# Supplementary material for: Compounds from plantar foot sweat, nesting material, and urine show strain patterns associated with agonistic and affiliative behaviors in group housed male mice, Mus musculus
Source: PLoS One. 2021 May 14;16(5):e0251416. doi: 10.1371/journal.pone.0251416 (PMC8121354; doi:10.1371/journal.pone.0251416)
Supplement: Attachment — Submitted filename: ReviewerComments20201202FINAL.docx [file pone.0251416.s024.docx]

*We humbly thank the reviewers for the comments below. We feel the suggested edits improve the both the scientific quality and reader comprehension of this manuscript. Each individual point has been addressed below.*

# Reviewer #1:

In this study, Barabas and colleagues collected body odours (urine, saliva, and plantar sweat, as well as nest odours collected from nesting material) from 3 mouse strains, in an attempt to provide a factual basis to the empirical observation that reusing bedding material reduces aggression (through olfactory signals) upon cage changing in mouse husbandry. Compounds were identified by GCMS and their abundance correlated to various behaviours (mostly pertaining to social/aggressive interactions). Some odour profiles were correlated with affiliative behaviours. Please note, the manuscript would have been clearer if the figure legends had featured together out of the main text body.

*Thank you for the feedback. We followed the submission instructions of the author guide so we cannot alter how legends and figures are presented in the main text body.*

Improvement of lab animal welfare is hugely important and it is important to promote practices that lead to happier animals, for ethical and scientific reasons (to limit the effect of stress on the physiology we study). But one could ask: if reusing soiled litter has been sufficiently shown to work (ref 12), do we need to know why? I am being provocatively pragmatic, but perhaps this point should be argued in the paper.

*As suggested, further justification has been added in the introduction, see lines 85-89 (clean version)*.

This study is well written and there are some very positive and interesting elements (e.g. the GC-MS analysis of cardboard mixed with bedding material is a great idea, for the study of body odours in nests). I also warmly welcome the fact that the authors have openly been forthright and discussed potential problems/limitations (e.g. use of pilocarpine).
My main issue is that the study is merely correlative and based on a number of assumptions. The authors have not claimed otherwise and this is reflected in the title (‘contain compounds associated with affiliative behaviors’) and rest of the manuscript. But what to make of this data if we don’t compare the efficiency of the soiled bedding material (and the olfactory signals it contains) in preventing aggression between cagemates? Perhaps some of strains have different olfactory performance? Instead of the putative pheromones, these molecules could simply be metabolites reflecting the product of differences in physiology and microbiota between the 3 strains tested. We cannot (and the authors have exerted restraint in their write up) assume any causality between the VOC signatures and behaviours. This lack of functional data severally limits the message/data interpretation.
Was this the best way to test the hypotheses laid out in the introduction? I cannot help thinking that if I had wanted to address those questions, I would have envisaged a study where soiled nesting material would be used to investigate modulation in aggression/affiliative behaviour. And then tested the odour signature. Otherwise the correlations are not grounded in any factual reality.

*Yes, it is possible that differences in strain compounds could be due to metabolic, microbiome, and/or unidentified genetic differences. However, the intent of this study was to serve as the first step of a framework laid out to identify pheromones in plantar sweat (Wyatt T. Semiochemicals: Pheromones, Signature Mixtures and Behaviour. In: Nielsen BL, editor. Olfaction in Animal Behaviour and Welfare. Boston, MA: CABI; 2017. pp. 36–38.) and whether there are strain differences in the types of compounds produced; the intent was not to examine whether soiled nesting material reduces aggression. Before a casual effect is tested, it is first necessary to examine what types of compounds are present in nesting material and then to examine whether there is a quantifiable behavior difference associated with a key molecule(s). This rationale has been added to the end of the introduction to further clarify the goal of the current project (lines 105-110, clean version ).*
Abstract: ‘This supports nest transfer as a recommended practice during cage change.’ … I am not sure this is right, how do the correlations described here support the practice??

*Conclusion statement has been rephrased.*

Title: I do love a joke and I am in favour in making scientific literature more exciting, but I am afraid I am not sure ‘friendship stinks’ contributes to a good informative title for a scientific article (in fact might it be slightly misleading?). I would just point out that (a) there is no indication these odours smell bad (perhaps they might even be attractive) and (b) the evidence between these odour and affiliative behaviours is merely correlative.

*We have changed the manuscript title.*

Introduction: it is a matter of opinion, but the mention of pheromone insinuates that the compounds identified will have pheromonal properties and causally affect behaviour (and this study does not show this). Even if the odours identified reduced aggression, this study does not show the compounds are pheromones, they could just be familiar odours.

*We did not intend to imply that what we were measuring were in fact, pheromones. Pheromones were only mentioned in order to give an overview of olfactory communication, and as an example of what may be held in nesting material. This section has been clarified.*

- Dominance: any anecdotal observation that the tube test correlated to other measures (e.g. small wounds, scruffy appearance)?

*Anecdotally, SJL mice with less wounding seemed to perform worse in the tube test. However, we did not formally test this so have not included this in the discussion.*

- Role of pilocarpine: Is there any evidence/data/comparison of VOC profiles with non pilocarpine treated individuals? (even in other types of body fluids) are there individual differences in pilocarpine response (ie differences in physiology linked to social rank/anxiety status etc)

*From what we could find, the only VOC profile comparison was done in human subjects either subjected to pilocarpine, or exercise to promote excess sweating. Whether this is comparable to mice is debatable, since mice do not sweat in the same way humans do to thermoregulate, but this has been added to the discussion. There is also documentation of individual variation in pilocarpine success for treating dry mouth, but factors behind this variation do not seem to be known. This has also been added as well as the volume range of saliva samples in this study.*

- Plantar sweat (line 69 and throughout) : I wonder whether the secretions were specifically that of a specific gland (producing that oily substance mentioned) or the product of all sweat glands on the injected foot area.

*The secretions from mouse foot pads are known to specifically come from eccrine sweat glands. This has been added to the text.*

- I am not sure the risk of contamination with fecal odours has been adequately discussed.

*It was mentioned in the discussion that fecal odors could be present in the nest material (line 310, clean version). However, we are unsure if this comment references specifically the contamination of nest material. Since we did not measure fecal odors in these samples, we do not feel comfortable speculating their role on the behavior observed here.*

*To avoid contamination with fecal odors, the methods describe how the surface of the foot and the anesthesia chamber were cleaned with ethanol before sweat and saliva were collected, respectively. Urine was also collected from surfaces that either never held feces or were cleaned with ethanol before the mice were placed on them.*

- Affiliative behaviour: Since this is central to this work, please ensure you provide a clear definition of affiliative behaviour (in the intro) and of the behaviours that belong to this category. I found examples in Fig 7 legend, but I believe this should be made clearer in the text.
*A definition and examples of affiliative behavior have been added to the introduction (line 66-68, clean version).*

Comments:
Table 1: browsing through the compounds detected, I found a couple of surprises, I checked that methyldihydrojasmonate has also been detected in human saliva (DOI: 10.1007/s10886-010-9846-7), however can the explain why limonene can be detected in mouse saliva?

*Limonene is a common mammalian metabolite, and it has been detected previously in human saliva (Huda AL-Kateb et al 2013 J. Breath Res.* ***7*** *036004). The methods do vary in these human papers, so that likely influences the chance of detection. However, the methods in our study are based on the first, where limonene wasn’t found. Perhaps it also depends on geographic region and local microbiome as limonene is produced by microorganisms as well (*[*https://doi.org/10.1080/10408440802291497*](https://doi.org/10.1080/10408440802291497)*; Table 2)*

Line 61: the statement ‘In contrast, little is known about pheromones that may promote affiliative behaviors among male mice’. Is this compatible with line 65 ‘Even though mice are not known to produce affiliative odors’?

*The latter has been reworded.*

Line 76: should it be ‘aim’ (singular)?

*This typo has been fixed.*

Line 83-85 (working hypothesis): this was not tested.

*This line has been edited to not assume causality.*

Line 91: to be more precise, add ‘cage per strain’ after n=8?

*Added*

Line 317-318: it has been reported that tube test hierarchy assessment after one measure does not provide a reliable indicator (doi: 10.1038/s41598-018-24624-4)

*Unfortunately, the referenced study was published while we were running this project. The citation is in the manuscript and we acknowledge that our tube test protocol may not have been the best method.*

Line 358 : missing punctuation

*Added*

Line 366: the discussion on the consequences of pilocarpine use is very welcome.

*Thank you for this comment.*

Line 470: pathogens/pathogen agents instead of agent?

*Edited*

Line 472-473: traditional control? The inter strain comparison does not require a control (what would it be?!?), what does this have to do with being an exploratory study? Consider rephrasing.
Line 486: I assume mentioning experimenter’s sex is in reference to studies demonstrating the impact on lab rodents (e.g. Sorge et al 2014). Should the rationale/reference be mentioned?

*This manuscript participated in beta testing for the ARRIVE 2.0 guideline. Both points were recommended for inclusion to improve the consistency of reporting. Regardless, a clarification to the statement has been added.*

Line 495: partially sighted or blinded to treatment?

*Edited*

# Reviewer #2:

Previous studies have shown that transferring soiled nest material when groups of male mice are transferred into a clean cage can reduce their aggression. The main aim of this study was to quantify volatile organic compounds (VOCs) in nest material soiled by male mice, identify the likely sources of nest odor compounds (from urine, saliva or plantar glands), and establish whether differences in aggression levels between strains correlate with levels of different VOCs (or combinations). A second aim was to determine the relationship between VOC profiles and mouse social behavior (more specifically, aggressive and affiliative behavior), particularly to test the hypothesis that VOCs in soiled nest material and plantar gland secretion (here referred to as sweat) reduce aggression and promote positive (affiliative) social interactions.

The manuscript contains some potentially important and novel data, particularly providing the first chemical analysis of nest material soiled by male mice and their plantar gland secretions. However, there are some fundamental issues with the experimental design and analysis that need to be addressed before it is possible to review the findings and conclusions of the study. As such, my review focuses only on these issues, together with a suggestion for improving the way that the findings are presented to make the study much more accessible to readers.

The study design uses eight replicate cage groups of 5 males for each of three inbred strains, selected due to differences in aggressiveness and because B6 is the most commonly used inbred mouse strain. Analyses confirm that the three strains showed highly significant differences in behavior and in VOC profiles for all three odor sources (nest, sweat and urine). Subsequent analyses then attempt to look at the relationships between VOC profiles for each odor source per cage and group social interactions, first using principal components analyses to reduce the number of variables and take into account correlation between individual VOCs or behaviors. However, to analyse correlations, the data are pooled across strains and strain is not included in the analyses. This is inappropriate – the replicates are not independent and drawn randomly from a population of mice, but instead represent replication of three independent genotypes. As strains were picked a priori for differences in social behavior, and (from previous work) differences in VOC profiles are expected between genetically distinct strains, correlations could simply be due to strain differences without implying any direct relationship between behavior and VOCs (just as genetically distinct strains differ in many other characteristics). Including strain in the analysis will address whether variation in VOC profiles between cages relates to differences in aggression or affiliative behaviour within and across genotypes. Currently, it is not possible to see how much affect this might have on analyses as strain is not shown in figures showing correlations (Fig 3, 4), but this is essential information. There are a number of ways to achieve this, but one possibility might be to use mixed effect models, which would also allow block to be included as a random factor (as in other analyses) and allows for some missing data without have to exclude all data for a particular cage (but this is just a suggestion). As the study is looking for any differences in VOC PCs that relate to either of two different measures of behavior, p values also need to be corrected for the multiple comparisons that essentially all address the same question (is there a relationship between behavior and VOC profile?), but I couldn’t see any mention of this in the statistical analysis section.

*Thank you for these comments. The analyses have been redone to include strain in the main PC models and mouse batch number (block). Correction for multiple comparisons has been done using the Bonferroni sequential method which is particularly useful for “areas that are in their infancy or those that are underexplored ,” (DOI:10.3233/NRE-130893).*

It is unclear in places how repeated measurements have been taken into account in analyses (ie where more than one data point was used per cage). For example, for the analysis of nesting behavior and other behaviors across the week (lines 255-268) the n size is given as 72 or 67 degrees of freedom for the error term, but this is surely incorrect. The n size was 24 cages, with repeated measures on 3 days. Consider using a repeated measures GLM with day as a within-subjects factor.

*In the models that analyze behavior for each study day, cage, nested in strain, is included as random factor. We did have an oversight and originally forgot to include it in the two nesting models, which has been corrected.*

I would also strongly encourage the authors to rethink the way that results are presented so that the findings are much more accessible to a reader – while this study is within my area of expertise and fundamental interest, I found the multiple statistical analyses presented in the text to be impenetrable and impossible to follow without drawing out my own summary tables. It would be a massive shame to put all this effort into the study but then have very few readers able to read and understand it. Could I suggest that analyses are provided in Tables so that the text focuses on summarising the main patterns of relationships from the perspective of the key question being addressed here – whether behavioural variation in aggression or in affiliation between groups is significantly related to VOC profile of nest material and/or specific odor sources, and which components might then be of particular importance. A diagram summarising key strain differences might also help.

*Thank you for this comment. Several tables have been added to the main text and supplementary data to present statistical values. We also included a strain summary diagram at the end of the results. We appreciate this feedback to help make our manuscript easy to understand.*

Given that the focus of the study is on the odors in nest material that may reduce aggression between males, this gets lost in the Discussion section. I would suggest that the authors make sure that this is the focus and include an appropriate critique of what conclusions can be drawn about the influence of VOCs on behavior from the patterns of correlation found (particularly across a small number of strains) and what further evidence is needed. Other points could then be addressed secondarily.

*The discussion has been reorganized in an attempt to address this comment.*

Please note that the colors used to distinguish between strains in Fig. 2 are difficult to distinguish, particularly when printed out.

*Colors have been adjusted.*

Data abstracted from GC/MS analyses are included in supplementary material but it is not clear whether the raw data files on which these values are based have been submitted to a depository. I could not find reference to the availability of raw behavioral data but this will be necessary to meet the PLOS Data policy.
*Thank you for pointing this out. I misread the requirement and raw GCMS files are submitted as compressed zip files in the Supplementary Information. However, they were obtained with a proprietary software and can only be opened with an Agilent GC-MS instrument with ChemStation software.*

I hope these comments are helpful.

*Yes, very much so!*
