# Supplementary material for: Compounds from plantar foot sweat, nesting material, and urine show strain patterns associated with agonistic and affiliative behaviors in group housed male mice, Mus musculus
Source: PLoS One. 2021 May 14;16(5):e0251416. doi: 10.1371/journal.pone.0251416 (PMC8121354; doi:10.1371/journal.pone.0251416)
Supplement: Attachment — Submitted filename: ReviewerComments20210301.docx [file pone.0251416.s025.docx]

*We would like to start by saying thank you to the reviewers for providing such valuable commentary. Particularly, it is clear reviewer 2 spent a lot of time critiquing this manuscript and their insight truly contributed to strengthening this work and making it more comprehendible. We send our utmost gratitude for this effort.*

*Additionally, we have updated the manuscript with a verified structure for MW 154 compound detected in sweat samples.*

Reviewer #1

I thank and congratulate the authors for their work to improve this manuscript. This report is very interesting and I am looking forward to reading up on future developments on the question of mouse aggression and plantar sweat. I would however suggest not to forget other odorant sources (e.g. urine or saliva), volatile compounds evaporate and can evade detection, and rarely biologically work in isolation (ie they are naturally occurring as part of mixtures, even though the literature glorifies single molecules like PEA, TMT and the likes).

Comments on ‘clean version’,
- Line 594 : is there a typo in this sentence?

*This has been reworded*

- Line 635: were utilized?

*This has been corrected*

- Throughout: I am not sure p’s is the plural of p, yet it could be this inappropriate use of apostrophe might be the best abbreviation for ‘p-values’

*We have corrected “p’s” to “p values”.*

# Reviewer #2

I am happy that the authors have responded appropriately to my major concern that strain was not included in correlation analyses between VOCs from different sources and behaviors, and that sequential Bonferroni has now been applied to adjust p values for multiple comparisons. An oversight to include day where appropriate as a within-subjects factor to avoid pseudoreplication has been corrected. I also found that inclusion of the new Tables 3 and 4 was very helpful in showing the patterns found in the data analysis much more clearly, while new Figure 6 provides a useful summary from such a complex data analysis (though I didn’t understand the color coding, see also other comments on the figure below). My review therefore focuses on interpretation of the data analysis and conclusions drawn. Some specific suggestions for some rewording are provided at the end of this review.

*The color scheme has been explained in Figure 6.*

The focus of the study is on identifying candidate VOCs in nest material that might account for reduced aggression observed previously when soiled nest material is transferred between cages at cage cleaning. VOCs associated with affiliative behavior (allogrooming and group sleep) are also examined, although relevance to the reduction in aggression in response to soiled nest material isn’t very clear, given aggression and affiliative behavior do not show a simple negative relationship (as confirmed in this study). As only affiliative but not aggressive behavior features in the title and much of the conclusions, this needs some further explanation as I struggled to understand the relevance to the main issue addressed here.

*You are correct that altering aggression was our focus, but we did not know what to expect in terms of behavior diversity in the cage. Affiliative behaviors were included since previous work on laboratory mouse social behavior primarily does not take place in the home cage and little is published on what influences these behaviors in the laboratory and how that would relate to aggression. Thus, we did not want to risk overlooking social differences that may have been something other than agonistic behavior. Additionally, both promoting affiliative behavior and reducing aggression would benefit mouse welfare. This explanation has been added to the intro (line 70-76).*

The study is designed to look for VOC candidates that might have common effects in regulating aggression across mouse strains. I think it is important to clarify this underlying assumption, as differences between chosen strains in the odors used to regulate aggression, or in sensitivity to these VOCs, would result in a lack of association using this design. An example is mentioned in the Discussion (though not in this context) where strains differ in expression of a scent protein that stimulates aggression (lines 445-450), but nonetheless strain differences in expression do not correspond to differences in aggression but to strain lineage, which deserves further comment.

*This assumption has been acknowledged in the end of the introduction and more explanation has been added to the discussion (line 106-107; 450-452 ).*

Although not anticipated when selecting the three strains used, the study found that aggression within two of the strains was rare during the week-long study and common only in one of the strains (SJL). This is important information for interpretation of the findings but this only becomes apparent to the reader quite late in the Results section, from Figure 5. PCA analysis of behavior derived two components that distinguish the main strain differences, but it is important to refer to the data in Figure 5 to interpret these components given the major strain differences – currently the analysis is quite misleading (though I’m sure this is not intentionally so). Scores on PC1 (referred to as Aggression PC) are influenced not only by aggression and social investigation but also by high negative weighting to allo-grooming (not shaded in Table 2 but this has just as strong a weighting as others that are shaded). The highly significant difference between strains reported for this component (SJL > AJ > B6) is not because AJ has greater aggression than B6, which the text implies (Table 3, lines 191-192). Instead, the weightings on this component reflect greater aggression and social investigation in SJL compared to AJ and B6, but greater allogrooming in B6 than AJ or SJL (see Figure 5, leading to SJL > AJ > B6). This component thus discriminates the major differences in behavior between strains, but cannot be interpreted as reflecting aggression alone and the label Aggression PC is quite misleading. PC1 does not entirely explain behavioral differences between strains because group sleep shows a slightly different pattern (but accounts for less variance). The second component (referred to as Affiliative PC) reflects this strain difference in group sleep while being orthogonal to PC1 (which has already accounted for strain differences in aggression, investigation and allogrooming). Thus PC2 weightings reflect greater group sleep in B6 and SJL vs AJ while AJ also has lower allogrooming than B6 and lower aggression and social investigation than SJL so scores very low for this component. PCA merely finds the main axes of variation in the data – interpretation of the derived components and strain differences in scores requires inspection of the underlying behaviors shown in Figure 5, so it does not make sense to me to split up the analysis of behavior using PCA and the later section on strain and behavior.

Once the pattern of differences between strains is clear, one can then relate this in an appropriate way to VOC differences. As it stands, the implication of Table 3 and text is that aggression follows the pattern SJL > AJ > B6, so VOCs matching differences in aggression should be either SJL > AJ > B6 or SJL < AJ < B6. Instead, it is clear from Figure 5 that VOCs matching differences in aggression between strains should discriminate SJL from the other two strains and that “Aggression PC” is misleading.

*Thank you for these comments and we agree with your suggestion about moving the sections. The behavior models have been moved earlier in the results section (line 209). Regarding our original approach to the behavior PCA, we had used contribution criteria to select meaningful variables, which in Behavior PC1, excluded allo-grooming. After your comment, we realized that we neglected to include this criteria in the manuscript, but also, upon further investigation, we determined this criteria is more suitable for identifying influential observations/sample units, not variables. Therefore allo-grooming has been included in the behavior PC1 interpretation and have removed our “Aggression” and “Affiliative” labels to avoid confusion.*

With that in mind, nest PC1 scores differ between SJL and the other two strains. However, as data on levels of the main compounds that contribute to nest PC1 are not presented, we cannot see which components differ consistently between SJL and both other strains (consistent with the hypothesis that some VOC components match strain differences in aggression) – important as the difference here is only just statistically significant while the strain difference in behavior is substantial. Would it not be better to focus detailed descriptions on this specific nest PC1 component as the one that does correspond with the strain difference of interest while also relating to nest material known to have aggression reducing properties, rather than losing the key relationship here in all the detail of VOC profiles that do not match the hypothesis being tested? The behavioral analysis clearly shows that the pattern of neither allogrooming nor group sleep (referred to as affiliative behaviors) correspond to strain differences in aggression, which needs to be pointed out much more clearly since the point of discussing VOC patterns in relation to ‘affiliative behavior’ (which do not correlate across strains and cannot simply be grouped together) is very unclear.

I do think there are interesting and novel data here, but this is being lost in an over-complex analysis that seems to be leading to confusion rather than insight. I would really advise the authors to consider focusing first on the strain differences in behavior which are quite clear cut. The finding that SJL was the only strain to show appreciable aggression then changes the focus of analysis of VOC profiles and could be presented in a very straightforward way. Allogrooming and group sleep each show different strain patterns so it is not possible to simply group these together as ‘affiliative behaviors’ in relation to strain differences from these analyses, and authors need to avoid the implication that mice that are affiliative are therefore not aggressive. If the main focus of the study is on VOCs that might appease aggression, shouldn’t the title reflect the study findings in relation to aggression not in relation to uncorrelated affiliative behaviors? It is just a suggestion, but throughout I found this confusing.

The discussion is extremely long and interpretations throughout really need revisiting in light of the points made above concerning the interpretation of behavior PCs and their match to VOCs. As a suggestion, perhaps some of the more speculative aspects could be dropped so that the focus remains clearly on the findings of the study.
*Thank you for these suggestions. The previous behavior PC labels have been removed and the discussion has been streamlined. We fully admit that we were so focused on all of the details from these data that we lost track of our main message. Thank you for helping us see that as well as help focus our manuscript. The discussion now focuses specifically on nest PC1 and urine PC2 (since their strain patterns match that of observed behavior) and sweat PC1 (since sweat was central to our hypothesis).*

Some specific points, particularly in relation to conclusions from analyses
Abstract line 33: The differences according to strain do not ‘mask direct effects on behavior’ in these analyses. Including strain in analyses increased the likelihood of seeing overall correlations between individual VOC profiles and behaviors, by taking into account any differences in levels of behavior or VOC profiles between strains. Instead analyses showed that there were no consistent relationships between individual VOC profiles and behavior that were not accounted for by major strain differences.
*This line has been reworded.*

Abstract lines 34-37: As the data show that the two affiliative behaviors analysed were not correlated (thus would be better referred to separately), while differences in these behaviors between strains did not match differences in aggression, this seems to miss the main results and conclusions that can be drawn from the data.

*These lines have been reworded.*

Abstract line 37: The term ‘correlation’ is not appropriate here as this implies a continuous statistical relationship which was not shown with 3 strains. Strain differences in VOC patterns that match strain differences in behavior would be more appropriate as a conclusion, being appropriately cautious here as there is no statistical validity in just picking out the VOC profiles that match.

*This line has been reworded.*

Abstract line 42: ‘provide preliminary information about a potential connection between …..
*Several changes to the abstract were made however we are unsure what alteration the reviewer was requesting here.*

Intro line 66-68: As affiliative behavior is often not negatively correlated with aggression (more social animals generally show more of both), the relevance of affiliative behavior to the issue of appeasing odors in nest material needs more explanation. Does addition of soiled nest material result in increased affiliative behaviors as well as reduced aggression?
*More explanation on why affiliative behaviors are of interest has been added (line 70-76).*

Intro line 81: This sentence doesn’t seem to make sense. Do you mean the mechanism behind the reduction in aggression observed in response to used nest material?
*This line has been reworded.*

Intro lines 91-104: There needs to be some introduction to the choice of strains included in the study here, particularly when the next section is Results rather than Methods. Readers may not be familiar with these strains and what is already known about their aggressive / affiliative behavior.
*A brief strain overview has been added to the intro (line 102-104)*

Intro, last sentence: This would be more suitable for Discussion rather than introduction to this study.
*This line has been moved.*

**Results** (specific comments, see main comments above about strain differences and interpretation of PCA components)
Line 128: I would have found ‘Sample VOC profiles’ more helpful here (everything is a sample)

*This line has been reworded*

Line 133-134: I couldn’t see any indication in Table 1 for which compounds had been previously identified as mouse urinary compounds.
*An additional symbol has been added to denote urinary signals.*

Line 173: I would avoid the term ‘significant’ here as it implies statistical significance relative to random.
*This line has been reworded.*

Line 176: See main comments – I think giving these interpretive terms to the behavior PCs is misleading given the actual strain differences in the behaviors contributing to these components.
*These labels have been removed.*

Line 178: Again, there is no validity to using ‘significant’ here perhaps ‘considered important’. As indicated above, this really requires inspection of the individual component data for interpretation but this hasn’t been included.
*This line has been reworded.*

Table 2: ‘mediated’ and ‘escalated’ mean nothing to the reader – there is space in the Behavior column to include ‘aggression’.
*These labels have been clarified.*

Table 4: Given the importance of strain differences in these different behaviors for any interpretation, could you indicate in the table which strains are significantly different from which (as in Table 3? As none of the strain x day interactions are statistically significant, this could be simply stated in a footnote, giving appropriate space for showing the pattern of strain differences. As indicated in main comments, I think this section needs to come much earlier so that the PCA analyses and behavioral match to VOC patterns are interpretable. Given that aggression was so much stronger in SJL than in the other two strains, it would make more sense to match VOC patterns to this difference than to principal components that represent a much more complex pattern of differences between strains.
*This table has been restructured and this section was moved (line 209-236). Thank you, this suggestion greatly improves the flow of the paper.*

Line 277: In summary, SJL displayed substantially more aggressive behavior?

*This line has been reworded.*

Line 279 & Figure 6: B6 mice did not display more group sleep than SJL
*This line has been reworded and group sleep was removed from the figure. As mentioned above, the color coding has been explained in the Figure 6 legend.*

**Discussion**
Line 292: As indicated, Aggression PC does not only include aggression, but your data do show that only SJL showed appreciable aggression.

*This line has been edited to refer to aggression directly.*

Line 299: Some of the component weights in nest PC1 are negative (low levels would result in high scores)

*Negative weights on nest PC1 have been included in this line.*

Lines 297- 301, 315-330: Does the pattern of urine PC2 scores really match the differences in aggression (not shown – you could plot this)?

*Measures of correlation have been added between urine PC2 and aggression, as well as nest PC1 and aggression. (line 272-274)*

Line 334-335: Surely sniffing behavior denotes gaining olfactory information. I don’t think anyone working in olfactory communication would define this as ‘affiliative’ as it would depend on context, so more appropriate peer-reviewed literature should be cited here.

*The affiliative classification has been removed from this line. We wanted to be more general in regards to how sniffing is classified for mouse behavior. We don’t want to speculate further since a more in depth evaluation of different types of sniffing (snout/urogenital) and their motivation was not conducted.*

Line 336-339: This entirely depends on the assumption that strains do not differ in their use of and sensitivity to the same compounds. This does not appear to be discussed, but an example is given later where this does not appear to be the case. Lines 445-450 indicates that darcin is expressed more in mice of the C57 lineage, but I don’t think ref 60 looks at SJL or AJ mice. A paper by Cheetham in 2009 (Physiology & Behavior) looks at lineage differences in expression of this protein, but this doesn’t correspond to the hypothesis here since the protein is not produced in both other low and high aggression strains (including SJL and AJ).

*The darcin example was about strain specific expression, but it does not imply that sensitivity depends on expression. A reference to Kaur et al. 2014 has been added to this section. Admittedly, it uses BALB/c mice, but they are of the same lineage as AJ. Based on our literature searches, pheromone sensitivity has not been assessed in AJ or SJL mice.*

Line 497-499 Conclusion: It is hard to support this statement based on the current analysis. This really needs a much clearer demonstration of the alignment of strain specific VOC patterns and strain specific differences in the specific behaviors of interest.

*Additional analyses of high loading compounds on nest PC1, urine PC2, and sweat PC1 have been provided to justify this statement. (line 250-278; 282 (Table 5))*
